# Risk factors for peri-megaprosthetic joint infections in tumor surgery: A systematic review

**DOI:** 10.1051/sicotj/2024008

**Published:** 2024-05-30

**Authors:** Vasileios Karampikas, Panayiotis Gavriil, Stavros Goumenos, Ioannis G. Trikoupis, Anastasios G. Roustemis, Pavlos Altsitzioglou, Vasileios Kontogeorgakos, Andreas F. Mavrogenis, Panayiotis J. Papagelopoulos

**Affiliations:** First Department of Orthopaedics, National and Kapodistrian University of Athens, School of Medicine 41 Ventouri Street, 15562, Holargos Athens Greece

**Keywords:** PJI, Risk factors, Limb salvage, Megaprostheses

## Abstract

*Background*: Peri-megaprosthetic joint infections (PJI) in tumor surgery are complex and challenging complications that significantly impact the outcomes of the patients. The occurrence of PJI poses a substantial threat to the success of these operations. This review aims to identify and summarize the risk factors associated with PJI in tumor surgery with megaprosthetic reconstruction as well as to determine the overall risk of PJI in limb salvage surgery. *Methods*: A thorough examination of published literature, scrutinizing the incidence of PJI in tumor prostheses after limb salvage surgery was done. Research studies that documented the incidence of PJI in tumor patients who underwent limb salvage surgery, and explored the risk factors associated with the occurrence of PJI were deemed eligible. *Results*: A total of 15 studies were included in the analysis and underwent comprehensive examination. After the exploration of key parameters, several significant risk factors for PJI concerning the type of implant coating, surgical site characteristics, patient demographics, and procedural factors were recorded. *Discussion*: The findings underscore the need for a nuanced approach in managing tumor patients undergoing limb salvage surgery and megaprosthetic reconstruction, with emphasis on individualized risk assessments and individualized preventive strategies.

## Introduction

The evolution of megaprostheses has markedly assisted in the reconstruction of large bone defects subsequent to the resection of bone tumors or soft tissue tumors invading bone for optimal function of the limb [1]. Peri-megaprosthetic joint infections (PJI) are many challenging complications that can occur following the use of a megaprosthesis in limb salvage surgery and may result in severe consequences [2]. With megaprosthetic reconstruction after tumor resection, the mean rate of PJI of a megaprosthesis is approximately 10% after the primary procedure, while it can be up to 60% after revision operations [3, 4]. Immunosuppression resulting from chemotherapy and radiation therapy, the presence of a substantial anatomical dead space after tumor resection, the absence of soft tissue structures for ideal wound coverage, extended operating hours, and mega implants are several significant factors that contribute to a high risk of PJI [3, 5].

Tumor patients with PJI after limb salvage surgery and megaprosthetic reconstruction often require staged revision surgeries and long-term intravenous antibiotic therapy; PJI-delayed adjuvant tumor treatments deteriorate patients’ quality of life and remaining life. In approximately 20% of cases, PJI of oncological prostheses leads to failure of the reconstruction or amputation of the limb [6]. Current therapeutic approaches for PJI include debridement-administration of antibiotics-irrigation-implant retention (DAIR), megaprosthesis revision (one or two stages), arthrodesis facilitated, and in select cases such as significant bone defect, lack of a bacterial isolate, and/or local tumor recurrence, amputation. The available clinical data pertaining to the outcome of these interventions for the management of the PJI are limited [7]; the prevailing method seems to be the two-stage revision operation [8]. Given the severe consequences associated with this severe condition, treatment strategies aiming to limit infection risk and optimize quality of life are of great importance.

This review article aims to comprehensively identify and summarize the risk factors associated with PJI in tumor surgery with megaprosthetic reconstruction as well as to determine the overall risk of PJI in limb salvage surgery.

## Materials and methods

The present systematic review was conducted in accordance with the guidelines of Preferred Reporting Items for Systematic reviews and Meta-Analysis [9]. A study protocol was designed and studies eligible for inclusion were identified through a thorough electronic systematic search of PubMed and Cochrane Library from February to April 30, 2023. The following search terms were utilized: ((“oncology” [All Fields]) OR (“tumor” [All Fields]) OR (“tumour” [All Fields]) OR (“neoplasm” [All Fields]) OR (“cancer” [All Fields]) OR (“limb salvage” [All Fields])) AND ((“prosthesis” [All Fields]) OR (“megaprosthesis” [All Fields]) OR (“endoprosthesis” [All Fields]) OR (“megaprostheses” [All Fields]) OR (“endoprostheses” [All Fields]) OR (“tumor endoprostheses” [All Fields])) AND ((“infection” [All Fields]) OR (“periprosthetic joint infection” [All Fields]) OR (“complication” [All Fields]) OR (“implant failure” [All Fields])). The search was restricted to articles published in the English language, with no limitations imposed on study types during the preliminary screening phase. Two authors independently performed the literature screening. Reviews and meta-analysεs were also analyzed aiming to expand the search for studies that might have not been detected by the electronic search methodology.

Studies that reported rates of PJI in tumor patients undergoing limb-salvage surgery and investigated risk factors for infection were considered eligible. Studies reporting outcomes of megaprosthetic reconstruction for non-oncologic conditions, case reports, editorials, and letters to the editors were excluded. For duplicates, only the most recent or most informative study was used.

**Figure 1:**
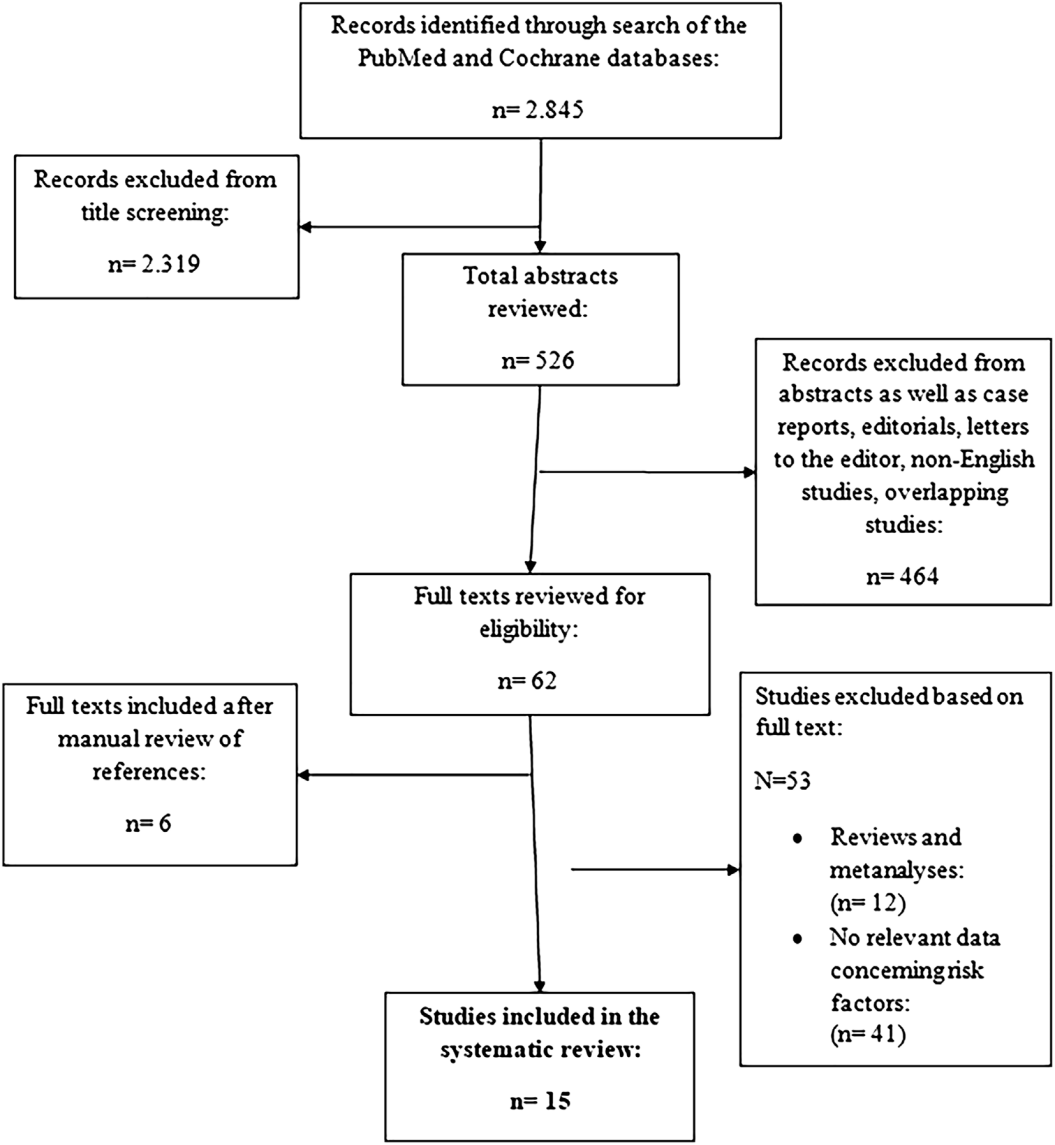
Flowchart and selection process of the included studies.

The results generated by the primary search algorithm and the stages of the selection process were delineated in a flowchart (Figure 1). Overall, a total of 2.845 studies were initially identified. Based on their titles 2.319 were excluded, leaving 526 studies for review. Their abstracts were subsequently assessed for relevance to our clinical inquiry, leading to the exclusion of 464 additional studies. Full-text articles were then obtained and thoroughly examined for the remaining 62 studies. Following a search of references, six full texts were added. Of these, 53 studies were further excluded due to the inability to extract relevant data concerning potential risk factors for prosthesis infection. Any discrepancies were solved after team consensus.

After exclusions, a total of 15 studies were left for review [2, 10–23]. All the studies included in the analysis underwent thorough evaluation, and relevant data pertaining to areas of interest were extracted and summarized (Table 1). Publication dates of the included studies ranged from 2005 to 2022 and sample sizes varied from 81 to 1240 patients.

**Table 1:** Characteristics and reported risk factors of the included studies.

Study	Patients (*n*)	Age (mean, years)	Follow-up (mean)	Infection rate (%, *n*)	Infection onset (mean time)	Type of infected prosthesis	Survival without infection	Risk factors	Risk factor analysis
Khakzad et al. [10]	83	–	3.9 years	16.8% (14)	141.4 months (knees); 64.6 months (hips); 8.2 months (shoulder)	4 PT, 5 DF, 2 PF, 2 PH, 1 TF	–	Primary tumor (*p* = 0.110)	*T*-test and Mann-Whitney test
								Knee arthroplasty (compared to hips)	
								Non-SC knees(compared to SC knees)	
								Higher CCI in PJI cases	
Streitburger et al. [13]	99	SC: 37 (5–82); Tit: 38 (7–71)	SC: 43 months	11.1% (11)	SC: median 4 months (0.5–17)	PF	SC: 90% tit, 83% at 5- and 10-year survival	Radiotherapy administration (*p* = 0.007)	Univariate analysis
			Tit: 95 months		Tit: median 11 months (2–55 months)				
Berger et al. [11]	115	53.4 (7.1–88.1)	7.6 years (1.3–13.3)	32% (35)	–	7 PF, 12 DF, 8 PT, 3 Pelvic, 4 PH, 1 DH	–	Primary sarcoma (both bone and ST)	Regression analysis
Jeys et al. [2]	1240	–	5.8 years (0.25–33.6)	11% (136)	Median 8.5 months in 96 PJI occurred within the first 2 years	18 PF (6.7%), 48 DF (10.3%), 57 PT (23.1%), 11 Pelvic (23%), 2 Humeral (1.1%)	85–90% and approximately 85% at 5-year and 10-year, varied according to implant site	Subsequent surgery	Cox regression analysis
								Tibial site	
								Pelvic site	
								Radiation therapy	
								Expandable prosthesis	
								Myeloma	
Gosheger et al. [23]	250	30.7 (7.4–80)	45 months (3–140)	12% (30)	–	8 PF, 12 DF, 7 PT	–	Extra-articular resection with DF (6.2-fold higher risk compared with intraarticular resection)	Chi-square test
Parry et al. [14]	394	32.4 (4–95)	54.9 months (2–136)	8.6% (34)	SC: median 7 months (0–22); Non-SC: 10 months (0–58)	SC: 1 PF, 1 DF, 8 PT, 1 other; Non-SC: 2 PF, 9 DF, 10 PT, 1 PH, 1 other	SC: 90.9% and 86.8%; Non-SC: 95.3% and 91.8% at 1-year and 5-year survival	Any adjuvant treatment	Cox regression analysis
								Proximal tibia site	
								Previous surgery	
Medellin et al. [15]	81	43 (12–86)	10.3 years (0–31.7)	19% (15)	50% of PJI at 2.1 months (0–7); 50% of PJI at a mean of 72 months (12–372)	81 TF	–	Combined proximal tibia arthroplasty	Cox regression analysis
								Poor range of movement (0° to 45° of flexion)	
								Previous surgery	
Hardes et al. [16]	98	SC: 19 (11–78)	SC: 17 months (5–120)	SC: 8.9% (5)	SC: median 8 months (3.5–19)	98 PT (56 SC)	SC: 90%	Wound healing disturbances (*p* = 0.040)	Univariate analysis
		Non-SC: 16 (11–69)	Non-SC: 111 months (3–212)	Non-SC: 16.7% (7)	Non-SC: 18 months (1–71)		Tit: 84% at 5-year survival	SC: prior intralesional surgery (*p* = 0.040) and operating time (*p* = 0.001)	
Allison et al. [19]	329	50 (11–90)	34 months (0.4–251)	13.1% (43)	–	10 hip prostheses, 24 DF, 9 PT	–	Radiation alone	Student *t*-test
								Chemotherapy alone	
								Sarcoma	
Peel et al. [20]	121	38 (14–86)	34 months (IQR 17, 80)	14% (17)	Median 541 days (IQR 41, 952)	13 Femoral replacements, 4 saddle prostheses	–	Increasing BMI	Univariate logistic regression
								Increasing operation time	
								Increasing post-operative blood transfusion requirements	
								Admission to ICU	
								Post-operative hematoma	
Morii et al. [22]	82	31.1 (5–86)	52.3 months (9-105)	17% (14)	10.9 months (<1–48) in 10 cases within first 12 months	8 PT, 6 DF	–	Skin necrosis	Cox proportional hazards modeling
								Surface infection	
								Resection of ≥3 heads of quadriceps muscle in DF	
Dhanoa et al. [17]	105	25 (7–89)	32 months minimum	12.38% (13)	9 days to 63 months	2 DF, 6 PT, 1 DT, 3 Pelvic, 1 DH	–	Proximal tibia site	Logistic regression model
								Pelvic site	
								Pre-operative hospitalization ≥48 hours	
								Subsequent surgery	
								Co-morbidities	
								Transfusion of >2 RBC units	
Funovics et al. [21]	166	49.8 ± 20.1 (5.9–84.3)	47 ± 67 months (0–365)	7.2% (12)	39 ± 60 months (0–167)	12 PF	95.9%, 89.2%, 89.2%, and 77.8% at 1, 5, 10 and 20-year survival	Primary tumor	Survival comparison with log-rank test
								Cemented fixation	
								Additional pelvic reconstruction	
Mavrogenis et al. [18]	1161	31.2 (7–80)	9 years (3–20)	8.6% (100)	3.7 years (0.5 months to 19 years)	10 PF, 61 DF, 27 PT, 1 TF, 1 Extra-articular knee resection	88% at 10 years and 84% at 20 years	Cemented fixation	Survival comparison with log-rank test
								Bone metastasis	
Fujiwara et al. [12]	121	42.1 (7–84)	5.9 years (0.1–19.8; median: 4.2)	12% (14)	Within 2 years in 6 patients; >2 years in 8 patients	3 PF, 7 DF, 4 PT	5-year and 10-year 100% and 100% (TF), 98% and 89% (PF), 90% and 79% (DF), and 95% and 60% (PT)	STT invading bone	Cox proportional hazards, ROC curve
								Primary tumor	
								Previous surgery	
								Male gender	
								Operating time	
								STT	
								Radiotherapy	

SC, silver coated implant; Tit, titanium implant; IQR, interquartile ratio; PJI, peri-megaprosthetic joint infection; PT, proximal tibia; DF, distal femur; PF, proximal femur; PH, proximal humerus; TF, total femur; BMI, body mass index; ICU, intensive care unit; RBC, red blood cell; STT, soft tissue tumor; ROC, receiver operation characteristic.

Variables of interest included general study characteristics (e.g. authors, year of publication, study design, country of enrollment, level of evidence, and number of patients), patient demographics (e.g. age, gender), oncological diagnosis, type of prosthesis, PJI rate, bacterial isolates, prophylactic antibiotic regimens, megaprostheses survival without infection and risk factors for PJI.

## Results

Eleven of the included studies in this review were retrospective in nature, presenting outcomes related to PJI after tumor resection and megaprosthetic reconstruction [11, 12, 14, 15, 17–23]. Two studies specifically compared results between patients with silver-coated and titanium prostheses. The silver-coated group was prospectively examined in both studies [13, 16]. Additionally, one study conducted a retrospective and prospective analysis focused on a specific time point [2]. Importantly, only one study maintained a prospective follow-up of their study group [10]. A total of 4.445 patients were included in all studies with a mean age of 35.78 years (range, 4–95 years). The mean follow-up of the patients ranged from 17 months [16] to 10.3 years [15]. Five studies included megaprosthetic reconstruction for tumors of the femur, tibia, and humerus [10, 14, 17, 20, 23], while three studies included megaprosthetic reconstruction for tumors of the lower extremity. Two studies focused on the outcomes of proximal femoral replacement [13, 21], one study on the outcomes of the proximal tibia [16], and another study on the outcomes of the total femur resection and reconstruction [15].

The mean rate of PJI was 13.77%, ranging from 7.2% to 32% among the included studies. The most common types of megaprostheses that sustained an infection were proximal tibia and distal femur megaprostheses. Concerning tumor diagnoses, osteosarcoma, chondrosarcoma, Ewing’s sarcoma, giant cell tumor of bone, and metastatic bone disease were most frequently encountered. Nine studies included in their analysis the perioperative antibiotic regimen that was administered (Table 2) [2, 10, 13, 16–18, 20, 22, 23]. As for the reported bacterial isolates, the predominant causative agents for the infections were coagulase-negative *staphylococci* and *Staphylococcus aureus* (including methicillin-resistant strains); several infections were multimicrobial.

**Table 2:** Summary of published studies reporting on antibiotics regimens for perioperative prophylaxis.

Study	Antibiotics regimen	Duration of antibiotics administration
Khakzad et al. [10]	– Ampicillin/sulbactam (usually 3 × 3 g i.v.)	–
Streitbuerger et al. [13]	i.v. 3rd-generation cephalosporin Oral therapy with a second-generation cephalosporin	Postoperatively: i.v. antibiotics for 3–7 days Oral antibiotics until wound healing is achieved
Jeys et al. [2]	i.v. dose of a broad-spectrum cephalosporin	Pre-operatively
Gosheger et al. [23]	i.v. cephalosporin	Postoperatively: i.v. antibiotics for 3–7 days Oral antibiotics until wound healing is achieved
Hardes et al. [16]	i.v. 3rd-generation cephalosporin Oral therapy with a second-generation cephalosporin	Postoperatively: i.v. for 3–7 days Oral therapy until wound healing was achieved
Peel et al. [20]	i.v. vancomycin was included in the surgical antibiotic prophylaxis regimen in 55 patients 87 patients had ongoing oral prophylactic antibiotics with cephalexin	Postoperatively: Median duration of i.v. antibiotics prophylaxis was 4 days Median ongoing oral antibiotics administration was 6 days Total post-operative antibiotics prophylaxis was 11 days
Morii et al. [22]	–	Post-operatively: Antibiotics were given for >72 h
Dhanoa et al. [17]	i.v. cefuroxime 30 min before incision and post-operatively. i.v. vancomycin for allergic patients	Post-operatively: For 5 days
Mavrogenis et al. [18]	Adults: Cefuroxime 750 mg/8 h (1983–1987) Teicoplanin 400–600 mg/day (1987–2010) Amikacin 500 mg/12 h (2 doses) (1983–2010)	Post-operatively: Cefuroxime for 5 days Teicoplanin for 1 day Amikacin; 2 doses Ceftriaxone in children for 5 days
	Children (<30 kg body weight): Ceftriaxone 30 mg/day (1983–2010)	

Age has not been associated with increased risk for PJI in any of the included studies [2, 10, 12, 22]. Although, most of the studies that investigated gender as a potential risk factor for PJI found no correlation [2, 17, 22], one study reported that male sex was a significant risk factor [12]. Patients with various comorbidities and increased Charlson Comorbidity Index (CCI) [10], as well as those with increased bone mass index (BMI), experienced a higher PJI risk [20]. Although only one study concluded that metastatic bone disease was associated with a higher risk for PJI [18], in all other studies the patients diagnosed with primary bone or soft tissue tumors experienced a higher risk for PJI [10–12, 19, 21]. Soft-tissue tumors extending into adjacent bone were found to be a significant risk factor in one study [12], while myeloma was also reported in another study [2]. The primary tumor diagnoses of osteosarcoma or Ewing’s sarcoma did not demonstrate a correlation with worse survival rates without PJI when compared to other types of sarcomas [18].

The implementation of any adjuvant therapy for the treatment of malignancy was shown to increase the possibility of infection [14, 18]; specifically, four studies reported that radiotherapy was a significant risk factor for PJI [2, 12, 13, 19]. Chemotherapy has been associated with a higher risk for PJI in one study [19], while in others it did not seem to be associated with increased rates of infection [2, 11, 12, 15, 20, 22]. Tumor location and reconstruction in the tibia [2, 14, 17] and the pelvis [2, 17] notably increased the risk of PJI. However, the length of bone resection prior to megaprosthetic reconstruction has not been found to predict a higher risk of PJI in two studies that investigated this variable [11, 22]. Additional reconstruction of the pelvis in cases of proximal femur replacement was a significant risk factor for PJI in one study [21]. In another study, knee megaprosthetic reconstruction was related to an increased risk for PJI compared to hip megaprosthetic reconstruction [10]. In cases of distal femur replacement, the extra-articular resection of the tumor prior to reconstruction [23] and the resection of more than three heads of the quadriceps in order to achieve wider surgical margins were significantly related to an increased risk of PJI [22]. One study with total femur reconstruction identified a poor range of motion postoperatively (0°–45° degrees of flexion) as a significant risk factor for PJI [15].

The relationship between PJI and implant coating has been previously reported [10–16]. One study found that the use of non-silver-coated implants in megaprosthetic reconstruction was associated with an increased risk for PJI [10], while in two other studies, the risk for PJI was the same regardless of the use of silver coating [14, 15]. Although superior implant survival rates without infection at 5 years were achieved with silver-coated megaprostheses, statistical analysis did not identify the implantation of a titanium prosthesis as a risk factor for PJI [13, 16].

Previous surgery prior to limb salvage and megaprosthetic reconstruction was found to be a significant risk factor for infection in four studies [12, 14–16]. These previous operations consisted of soft-tissue tumor resection and femoral fracture osteosynthesis [12], as well as prior curettage [12, 16]. Additional surgical interventions were also found to be significant risk factors in two studies [2, 17]. The utilization of expandable prostheses in pediatric tumor patients [2] coupled with subsequent lengthening procedures has been related to a higher risk for PJI [17]. In contrast, a separate study revealed that revision surgery resulting from mechanical failures did not increase the susceptibility to infection [16]. An increased risk for infection was found for patients who experienced wound healing complications postoperatively [16, 20, 22], including wound necrosis and superficial infection [22], and postoperative hematoma formation [20].

Several procedure-related factors were investigated in the included studies. Three studies reported that increased operating time was a significant risk factor for PJI [12, 16, 20]; one study found that operation time over a cut-off value of 493 min significantly increased the risk of PJI [12]. Preoperative hospitalization >2 days [17], as well as admission to the intensive care unit [20] have also been related to higher rates of PJI. The need for blood transfusion [20] and transfusion of more than two blood units [17] were also found significant risk factors.

## Discussion

Peri-megaprosthetic joint infections are challenging complications in limb salvage surgery for musculoskeletal tumors, with several risk factors contributing to a high risk for PJI. We performed this review to comprehensively identify and summarize the risk factors associated with PJI in tumor surgery with megaprosthetic reconstruction as well as to determine the overall risk of PJI in limb salvage surgery. Our findings showed a multifaceted nature of PJI in megaprosthetic reconstructions in tumor surgery, with key factors contributing to infection including surgical site characteristics, patients’ demographics, and procedure-related factors. Although similar investigations have already been made clear in a number of relevant, well-published studies, we believe that listing and analyzing these up-to-date published studies in a well-designed review article with systematic as well as narrative methodology is didactive and educative, as well as useful in clinical practice for optimal decision making in patients’ management.

The diagnosis and management of PJI typically involves a comprehensive assessment, including clinical evaluation, laboratory tests, and imaging studies to accurately identify and address potential infections around the prosthetic joint [24–30]. It may lead to a significant decline in patient health status, prolonged hospitalization, and unfavorable functional outcomes and prognosis [31–33]. Novel surgical techniques and antibiotic regimens are also necessary to be implemented in order to successfully manage this complication [34–37]. In this systematic review, we attempted to investigate potential risk factors for PJI in tumor surgery with megaprosthetic reconstruction. These factors may be related to patient characteristics, comorbidities, and medical conditions due to the malignancy or the surgical technique.

Megaprosthetic reconstructions after tumor resection have shown higher rates of PJI compared to conventional arthroplasty [18, 38–40]. In our analysis, we found that surgery for primary tumors, male gender, long operation time, radiation therapy, previous surgery, tibial and pelvic site of reconstruction, wound healing complications, intensive care unit admission, blood transfusion, and prolonged hospitalization were significant factors that increase the risk for PJI in tumor surgery [2, 10–14, 16, 17, 19, 20]. Leukocytopenia and neutropenia resulting from chemotherapy, along with tissue damage by radiation therapy may significantly influence the occurrence of PJI. However, the association of these adjuvant therapies remains controversial [41]. Patients who received chemotherapy reported higher overall revision rates compared to those who did not [42]. Several studies conclude that chemotherapy is not related to higher PJI rates [2, 11, 15, 20, 22], while other studies report that chemotherapy is a significant risk factor for PJI in tumor surgery [14, 19]. Although not statistically significant, survival from infection of tumor prosthesis was slightly better for patients who received radiation therapy or chemotherapy compared to those who did not with a survival rate of 88% and 90% at 10 years, respectively [18]. In a large study including 1264 tumor patients who underwent limb salvage surgery, a PJI rate of 11% was reported. Radiation therapy was a significant risk factor, as 20.7% and 35.3% of the patients who had pre- and post-operative radiation therapy, respectively, experienced a PJI compared to 9.8% of the patients who did not [2]. These results are in accordance with other similar studies [12–14, 19].

Knee megaprosthetic reconstructions have been associated with a higher risk for PJI compared to hip reconstructions [10]. Extra-articular resection with distal femur replacement showed a 6.2-fold risk for PJI compared to intra-articular resection [23]. Cementless fixation and rotating-hinge knee implants might also increase the infection rate in distal femur replacement [43]. Higher risks for PJI are anticipated in pelvic and proximal tibia resections and reconstructions as it has been found to be a significant risk factor [2, 14, 17, 21, 44]. Especially in proximal tibia resections and reconstructions, the risk of PJI is higher because of challenges in adequate soft tissue coverage [16, 45]. Since the routine use of a gastrocnemius rotation flap became a standard technique, the infection rate reduced from 36% to 12%. This significant improvement in the occurrence of infection is attributed to the better muscle coverage achieved with the flap [46]. Another study reported that resection of >37% of the tibia and resection length of >12.5 cm are associated with a higher risk for implant failure due to PJI [47]. Long operation time, extensive exposures, and residual dead space may significantly increase infection rates in the pelvis [2, 17, 21, 47, 48].

Antibiotic-loaded cement is routinely used for implant fixation in megaprosthetic reconstructions. Nevertheless, the literature presents highly diverse findings concerning the correlation between infection rates and cemented or cementless fixation in tumor surgery [31]. Cemented fixation posed a higher risk of infection in two large-scale studies compared to cementless fixation [18, 21]. In contrast, cementless fixation of prostheses showed significantly better overall survival and survival to infection compared to cemented fixation in another study. In that study, survival to infection was 68% and 82% at 60 months for cemented and cementless fixation, respectively [49].

The efficacy and safety of silver as an antibacterial coating on implants in order to reduce the incidence of PJI and improve outcomes of treatment of PJI is a matter of study during the past years [50, 51]. A recent comprehensive meta-analysis indicated a relative protective effect of silver coating in PJI prevention in megaprosthetic reconstructions. In particular, overall infection in primary silver-coated and uncoated implants was 9.2% and 11.2%, respectively. Moreover, optimal results with silver-coated implants were obtained in proximal femur replacements [52]. Two studies showed better implant survival without infection at 5 years in silver-coated prostheses [13, 16]. The use of uncoated implants in knee megaprosthetic reconstructions after tumor resection increased the risk of infection in another study [10].

We see two limitations in this study. First, the majority of the included studies are retrospective (Level of Evidence III or IV), with not all-encompassing study variables. Second, there is significant variability in the statistical methods employed across the studies. Consequently, obtaining more secure and unequivocal results, as well as facilitating comparisons between potential risk factors, presented increased challenges. We acknowledge these limitations; however, we believe that the study design and inclusion of retrospective studies are useful for decision-making in clinical practice.

## Conclusions

The comprehensive analysis of risk factors for PJI in tumor surgery may contribute to a better understanding of the challenges associated with these complex procedures and the management of PJI. Key factors contributing to infection risk include surgical site characteristics, patients’ demographics, and procedure-related factors. The present systematic review emphasized the multifaceted nature of PJI in megaprosthetic reconstructions in tumor surgery. The identification of poor outcomes and treatment-related challenges further highlights the urgency for tailored interventions. The integration of individualized risk assessments and personalized preventive measures to enhance the success of megaprosthetic reconstructions in tumor surgery is paramount.

## Data Availability

Data are available on request from the authors.
